# Extracts Prepared from Feed Supplements Containing Wood Lignans Improve Intestinal Health by Strengthening Barrier Integrity and Reducing Inflammation

**DOI:** 10.3390/molecules27196327

**Published:** 2022-09-26

**Authors:** Mara Heckmann, Nadiia Sadova, Ivana Drotarova, Stefanie Atzmüller, Bettina Schwarzinger, Roberto Mauricio Carvalho Guedes, Paula Angelica Correia, Stefan Hirtenlehner, Christine Potthast, Gerald Klanert, Julian Weghuber

**Affiliations:** 1 Center of Excellence Food Technology and Nutrition, University of Applied Sciences Upper Austria, Stelzhamerstraße 23, 4600 Wels, Austria; 2 FFoQSI GmbH–Austrian Competence Centre for Feed and Food Quality, Safety and Innovation, Technopark 1D, 3430 Tulln, Austria; 3 Department of Veterinary Clinic and Surgery, Veterinary School, Universidade Federal de Minas Gerais, Belo Horizonte 130161-970, Brazil; 4 Agromed GmbH, Bad Haller Str. 23, 4550 Kremsmünster, Austria

**Keywords:** feed supplements, intestinal barrier, wood lignans, barrier integrity, intestinal permeability

## Abstract

Lignans are known to exhibit a broad spectrum of biological activities, indicating their potential as constituents of feed supplements. This study investigated two extracts derived from the feed supplements ‘*ROI*’ and ‘*Protect*’—which contain the wood lignans magnolol and honokiol (‘*ROI’*), or soluble tannins additional to the aforementioned lignans (*‘Protect’*)—and their impact on selected parameters of intestinal functionality. The antioxidant and anti-inflammatory properties of the extracts were determined by measuring their effects on reactive oxygen species (ROS) and pro-inflammatory cytokine production in vitro. The impact on intestinal barrier integrity was evaluated in Caco-2 cells and *Drosophila melanogaster* by examining leaky gut formation. Furthermore, a feeding trial using infected piglets was conducted to study the impact on the levels of superoxide dismutase, glutathione and lipid peroxidation. The *Protect* extract lowered ROS production in Caco-2 cells and reversed the stress-induced weakening of barrier integrity. The *ROI* extract inhibited the expression or secretion of interleukin-8 (IL-8), interleukin-6 (IL-6), interleukin-1β (IL-1β) and tumor necrosis factor α (TNFα). Moreover, the *ROI* extract decreased leaky gut formation and mortality rates in *Drosophila melanogaster*. Dietary supplementation with *Protect* improved the antioxidant status and barrier integrity of the intestines of infected piglets. In conclusion, wood lignan-enriched feed supplements are valuable tools that support intestinal health by exerting antioxidant, anti-inflammatory and barrier-strengthening effects.

## 1. Introduction

Plants and their secondary metabolites have been widely recognized as a valuable source of novel therapeutic compounds for the prevention and treatment of a broad variety of diseases. Most of the pharmacological effects of medicinal plants have been linked to secondary metabolites, such as flavonoids, alkaloids and terpenoids [1]. These bioactive compounds are not only of high interest in modern medicine for preventing and treating human diseases [2] but have also been successfully used in animal nutrition. As feed supplements, these compounds are used to improve nutrient absorption, exert antioxidant effects, reduce pathological stress in the gut and beneficially impact animal growth performance, potentially replacing the use of antibiotic growth promoters [3,4,5,6,7]. Lignans represent a promising group of bioactive compounds that are relevant constituents of food and feed supplements [8,9,10,11] and have a broad range of molecular structures and biological activities. Lignan backbones consist of two phenylpropanoid units that are occasionally glycosylated. Lignans can be found in most fiber-rich plants, including grains, vegetables, seeds and fruits, where they are involved in protection against diseases and pests, as well as plant growth control [12]. Plant lignans have been shown to exhibit numerous pharmacological properties, including anticancer [13,14,15], antitumor [16,17], anti-inflammatory [18,19,20,21], antioxidant [22,23,24] and antimicrobial [25,26] effects.

Magnolol and its isomer honokiol are bioactive lignans extracted from the bark of *Magnolia officinalis* and display multiple biological activities [27]. Products containing magnolol or honokiol have become available for use in animal feed and are usually used as feed additives in low concentrations. The literature indicates that these wood lignans improve animal health and performance by exerting a broad spectrum of pharmacological activities. For example, it has been demonstrated that the integration of magnolol into the diet improves the egg production, egg quality, intestinal histomorphology and intestinal mucosal barrier function of laying hens [28]. It has also been shown that magnolol and honokiol support intestinal health in mice. Enhancing intestinal anti-inflammatory capacities, elongating villus height and crypt depth and inhibiting intestinal epithelial apoptosis are some of the reported mechanisms [29]. Additionally, honokiol extract could improve nonenzymatic and enzymatic antioxidant defense systems and decrease the severity of diarrhea induced by castor oil in mice [30,31]. Using an intestinal mucositis mouse model, Xia and colleagues showed that magnolol protects the intestine by reducing body weight loss, reversing histopathological changes and preventing colon length reductions. Oxidative stress and inflammation were also suppressed by the enhanced activity of the antioxidant effectors superoxide dismutase and glutathione peroxidase, an increase in the concentration of glutathione, a reduction in the amount of nuclear factor kappa b (NF-κB) and the downregulation of certain proinflammatory cytokines [32].

In addition to lignans, polyphenolic compounds derived from wood are a major class of bioactive plant metabolites with numerous biological activities. Tannins are an important group of polyphenolic compounds and represent strong candidates to be used in feed and food. They are widely distributed in the plant kingdom, with high concentrations in leaves, bark, wood, seeds and unripe fruit [33]. Wood extracts are one of the major sources of industrially obtained tannins and are increasingly applied as additives in food and feed [34]. Among those extracts, the European chestnut tree (*Castanea sativa*) represents a relevant source of tannins, with vescalin, castalin, gallic acid, vescalagin, castalagin and ellagic acid being the main representatives [35]. Tannins are reported to possess a variety of health-promoting properties, including antioxidant, antimicrobial [36] and antidiabetic effects [37]. The addition of plant extracts that are rich in phenolic compounds to animal feed as an alternative to antibiotic growth promoters is therefore of high interest. In pigs, dietary supplementation with tannins has been shown to increase growth performance, modulate intestinal microbiota and prevent diarrhea [38,39]. Moreover, chestnut tannins can counteract the negative effects of heat stress on the intestinal function and the growth of broilers. It was shown that mucosal barrier function was strengthened by regulating tight junction protein expression and by reducing the expression of the inflammatory cytokines interleukin-6 (IL-6) and tumor necrosis factor α (TNFα) and the transcription factor NF-κB in the jejunum of heat-stressed broilers [40].

In the present study, we analyzed the two feed supplements *agromed^®^ ROI* (*ROI*) and *agromed^®^ Protect* (*Protect*) and derived extracts containing varying concentrations of the wood lignans magnolol and honokiol and of polyphenols, specifically, soluble tannins. *ROI* and *Protect* are commercial feed supplements for pigs and poultry and have been shown to improve essential performance parameters such as weight gain and survival rate. However, their mode of action remains mainly unclear. We hypothesized that treatment with these products would positively affect intestinal barrier integrity and initiate antioxidant and anti-inflammatory processes by reducing relevant signaling molecules such as reactive oxygen species (ROS) and proinflammatory cytokines. By implementing and combining different in vitro (Caco-2 and THP-1 cells) and in vivo (*Drosophila melanogaster*) models, we provided a novel approach to investigating the potential health-promoting effects of wood-based extracts. Additionally, we performed a feeding trial using piglets infected with the Gram-negative bacterium *Brachyspira hyodysenteriae*, which is the primary etiologic agent of swine dysentery, to evaluate the potential benefits of wood lignan-based products in animal nutrition.

## 2. Results

### 2.1. Magnolol and Honokiol Concentrations of Extracts Prepared from ROI and Protect

**Figure 1:**
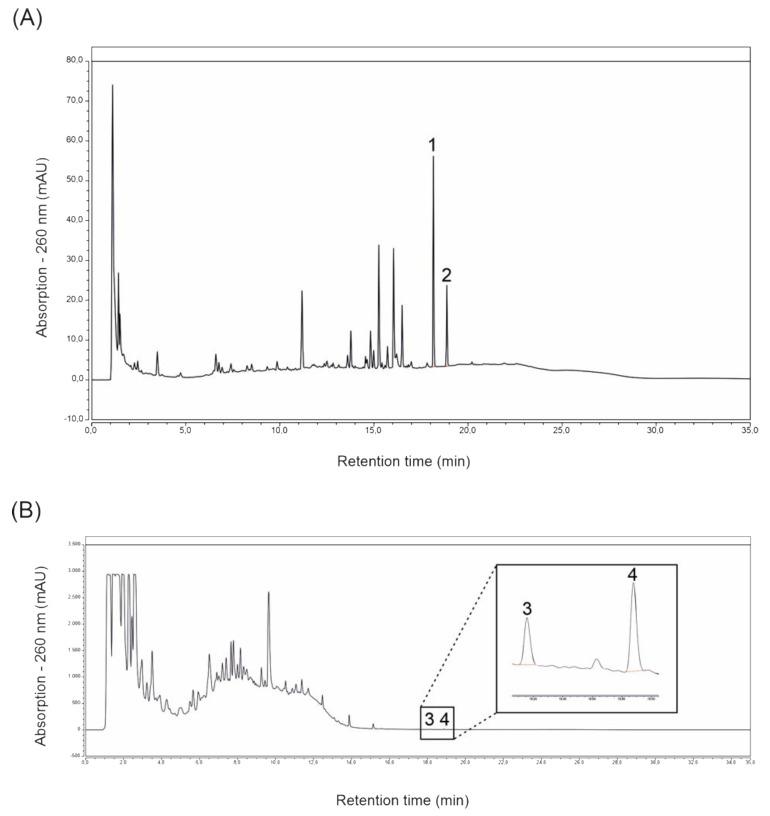
HPLC lignan analyses of *ROI* (**A**) and *Protect* (**B**) extracts at 260 nm. Identified peaks 1 and 3: honokiol; identified peaks 2 and 4: magnolol.

High-Performance Liquid Chromatography (HPLC) analyses of extracts prepared from *ROI* and *Protect* revealed the presence of the lignans magnolol and honokiol in both extracts. The main peaks of the *ROI* extract analysis were identified as magnolol and honokiol, with concentrations of 5.33 and 3.39 mg/L, respectively (see Figure 1A). *Protect* extract analysis revealed a magnolol concentration of 3.62 mg/L and a honokiol concentration of 0.45 mg/L. In contrast to *ROI*, Figure 1B shows that the lignans magnolol and honokiol were not the main compounds present in the extract prepared from *Protect*. This was expected, as *Protect* is enriched with polyphenols (soluble tannins) from *Castanea sativa*. The definite identity of these tannins has not been clarified so far.

### 2.2. Total Phenolic Content and the Antioxidant Capacity of Extracts Prepared from ROI and Protect

**Figure 2:**
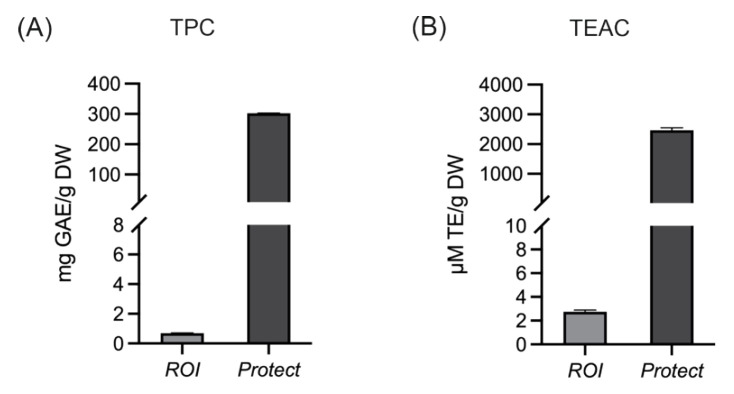
TPC (**A**) and TEAC (**B**) analysis of extracts obtained from *ROI* and *Protect*; the results are expressed as mg GAE per g DW and µM TE per g DW, respectively. Error bars are based on the standard error of the mean (SEM) of 3 replicates.

The phenolic content and antioxidant capacity of wood lignan-based extracts prepared from *Protect* and *ROI* were characterized via total phenolic content (TPC) and Trolox Equivalent Antioxidant Capacity (TEAC) analyses. As mentioned before, the extracts differ in their levels of polyphenols. This difference is shown in Figure 2. The total phenolic contents of the extracts prepared from *Protect* and *ROI* were 203 and 0.7 gallic acid equivalents (GAE) per g dry weight (DW), respectively. Consequently, *Protect* also showed higher TEAC values, with ~2.5 mM Trolox equivalents (TE) per g DW, compared to 2.8 µM TE/g DW for *ROI*.

### 2.3. Cytotoxicity of extracts prepared from ROI and Protect

**Figure 3:**
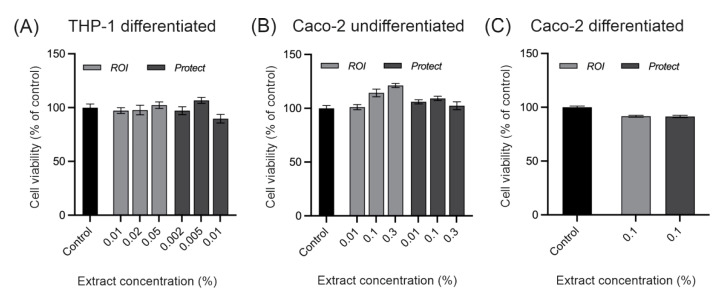
Cytotoxic assessment of *ROI* and *Protect* extracts in differentiated THP-1 cells (**A**), undifferentiated Caco-2 cells (**B**) and differentiated Caco-2 cells (**C**). Cells were grown in 96-well plates at 5 (**A**), 7.5 (**B**), or 8.25 (**C**) × 10^5^ cells/well and treated with the *ROI* and *Protect* extracts at the indicated concentrations for 24 (A) or 4 h (**B**,**C**). Error bars are based on the SEM of 8 (**A**), 10 (**B**), or 11 (**C**) replicates.

The cytotoxicity of the *ROI* and *Protect* extracts was evaluated in Caco-2 and THP-1 cells. Values above 90% were considered nontoxic and suitable for in vitro experiments. Figure 3 shows the viability of THP-1 cells and undifferentiated and differentiated Caco-2 cells after the addition of the *ROI and Protect* extracts. The THP-1 and Caco-2 cells were treated for 24 and 4 h, respectively. Preliminary data indicated that differentiated THP-1 cells are more sensitive to the effects of toxic compounds than Caco-2 cells. Thus, lower extract concentrations were applied to the THP-1 cells. As demonstrated in Figure 3, none of the tested extract concentrations showed cytotoxic effects. Based on these findings, 0.05% *ROI* and 0.005% *Protect* extract were chosen for the THP-1 inflammation assay. For the ROS assay using undifferentiated Caco-2 cells, a concentration of 0.3% was chosen for both extracts. In differentiated Caco-2 cells, a concentration of 0.1% for both extracts was found to be non-toxic. Therefore, only concentrations lower than 0.1% were applied for the intestinal barrier integrity tests, which were performed with differentiated Caco-2 cells.

### 2.4. Protect Extract Reduces Intracellular ROS Production

**Figure 4:**
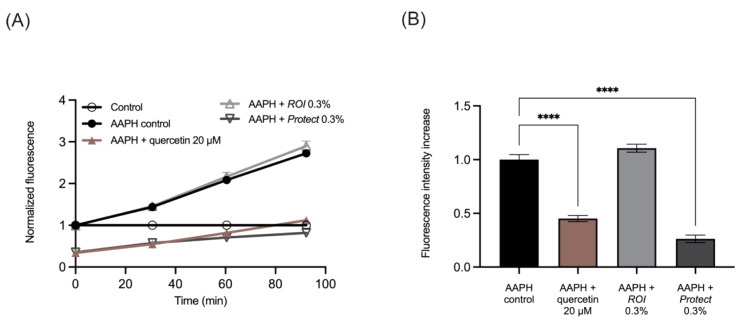
Measurement of ROS levels in Caco-2 cells. (**A**): Increased DCF fluorescence signal over time and normalized to the control. (**B**): Changes in DCF fluorescence signal are shown as normalized slopes based on 4 different timepoints measured after the addition of 500 µM AAPH. Cells were grown in 96-well plates overnight (1.5 × 10^5^ cells/well), and then, treated with *ROI* or *Protect* extracts or quercetin at the indicated concentrations for 20 min. ROS generation was determined using the cell permeant reagent H_2_DCF-DA. Error bars are based on the SEM of 6 replicates. ****: *p* < 0.0001.

The cell-permeable reagent 2′,7′-dichlorodihydrofluorescin diacetate (H_2_DCF-DA) was used to determine whether *ROI* and *Protect* extracts affected ROS generation in Caco-2 cells. Figure 4A shows an increase in fluorescence over time in response to the addition of AAPH. This increase was similar to the effect of 0.3% *ROI* on cells*;* thus, *ROI* did not affect ROS generation. *Protect* (0.3%) and the bioactive control quercetin (20 µM) reduced the levels of DCF fluorescence during the experimental period. The slopes between the timepoints were then calculated and normalized to the stressed control containing AAPH, which is shown in Figure 4B. In summary, similar to quercetin, *Protect* significantly lowered ROS generation in Caco-2 cells, while *ROI* did not affect ROS generation compared to the control.

### 2.5. ROI and Protect Extracts Decrease the Expression Levels and Secretion of Pro-Inflammatory Cytokines

**Figure 5:**
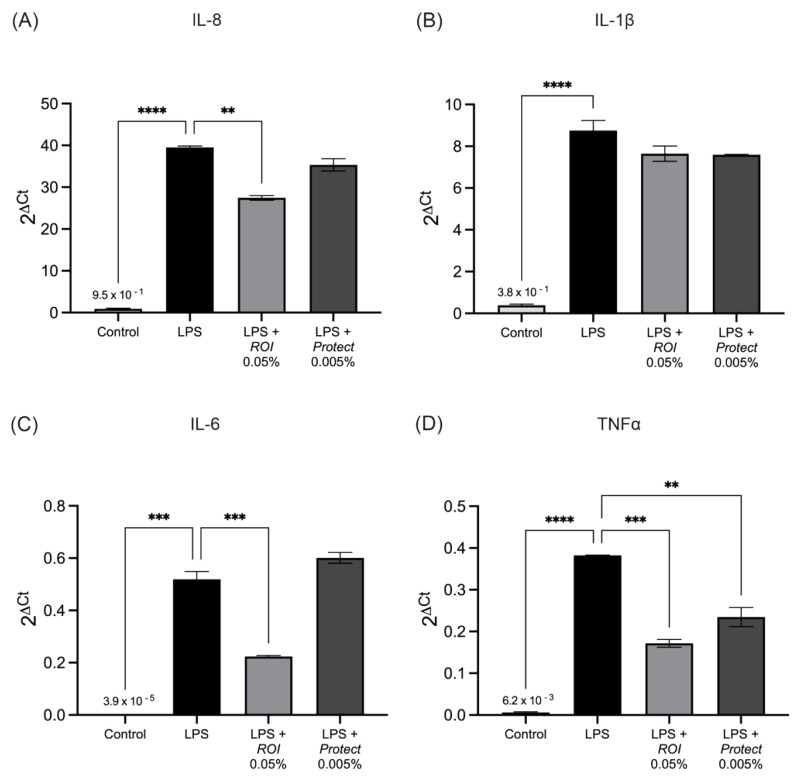
The mRNA expression levels of IL-8 (**A**), IL-1β (**B**), IL-6 (**C**) and TNFα (**D**) in LPS-stimulated THP-1 cells following 24 h of treatment with *ROI* and *Protect* extracts. Cells were grown and differentiated in 6-well plates (5 × 10^5^ cells/mL), stimulated with LPS (250 ng/mL) and treated with *ROI* and *Protect* extracts at the indicated concentrations for 24 h. Error bars are based on the SEM of 2 biological and 2 technical replicates. **: *p* < 0.01, ***: *p* < 0.001, ****: *p* < 0.0001.

We examined the effects of the *ROI* and *Protect* extracts on the lipopolysaccharide (LPS)-induced production of interleukin-1β (IL-1β), interleukin-8 (IL-8), IL-6 and TNFα in THP-1 cells. Figure 5 shows significant upregulation of IL-6, IL-1β, IL-8 and TNFα levels in response to LPS administration. The addition of *ROI* significantly decreased the mRNA expression of IL-8, IL-6 and TNFα in comparison to the control. *Protect*, at the used dosage, only significantly reduced the mRNA expression of TNFα.

**Figure 6:**
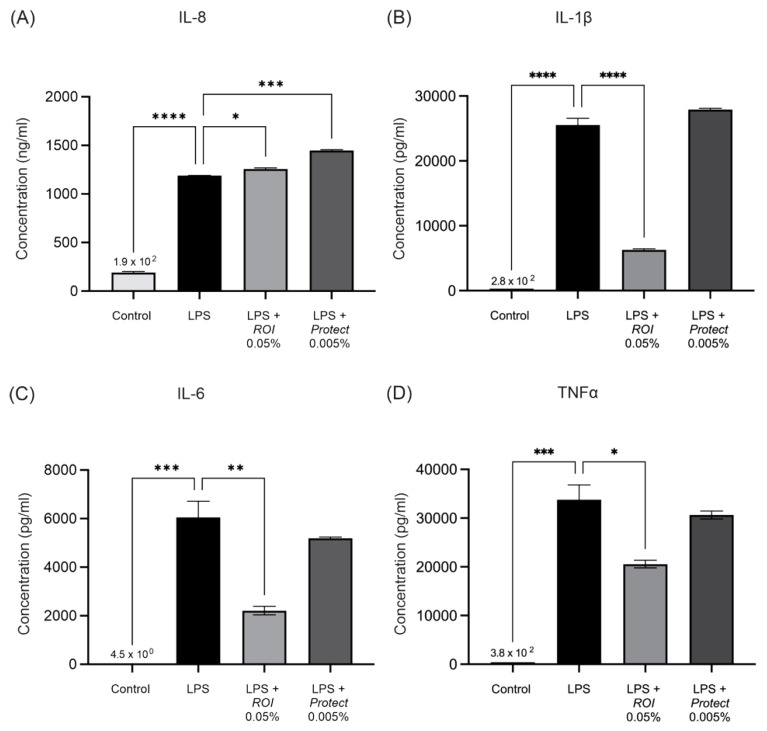
The concentrations of the cytokines IL-8 (**A**), IL-1β (**B**), IL-6 (**C**) and TNFα (**D**) in THP-1-cell culture supernatants were measured via multiplex immunoassay. Cells were grown and differentiated in 6-well plates (5 × 10^5^ cells/mL), stimulated with LPS (250 ng/mL) and treated with *ROI* and *Protect* extracts at the indicated concentrations for 24 h. Error bars are based on the SEM of 2 biological and 2 technical replicates. *: *p* < 0.05, **: *p* < 0.01, ***: *p* < 0.001, ****: *p* < 0.0001.

In accordance with the mRNA expression levels detected using RT-qPCR, cytokine secretion measurements revealed significantly elevated concentrations of IL-6, IL-1β, IL-8 and TNFα in the cell supernatant upon LPS-stimulation (see Figure 6). *ROI* significantly decreased the concentrations of IL-1β, IL-6 and TNFα, and slightly increased the IL-8 concentration in the supernatant of LPS-stimulated THP-1 cells. Treatment with *Protect* significantly increased the concentration of IL-8 in the supernatant, but did not significantly alter the IL-1β, IL-6 or TNFα concentrations.

### 2.6. Protect and ROI Extracts Stabilize Caco-2 Epithelial Monolayers

**Figure 7:**
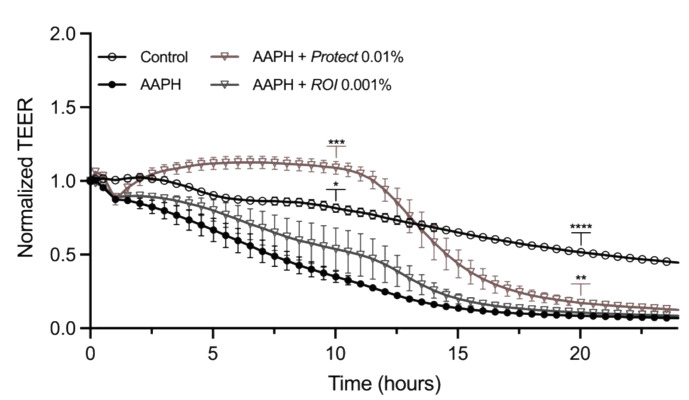
Normalized TEER values of differentiated Caco-2 cell layers treated with *ROI* and *Protect* extracts. Caco-2 cells were differentiated on transwell inserts, stressed with 5 mM AAPH and treated with *ROI* and *Protect* extracts at the indicated concentrations for 24 h. TEER values were normalized to the starting TEER value of each sample. Error bars are based on the SEM of 3 replicates. *: *p* < 0.05, **: *p* < 0.01, ***: *p* < 0.001, ****: *p* < 0.0001.

The transepithelial electrical resistance (TEER) value of a differentiated Caco-2 cell layer represents intestinal barrier integrity and permeability. To determine the potential positive effect of *ROI* and *Protect* extracts on intestinal barrier function, differentiated Caco-2 cells were stressed with 2,2’-Azobis(2-amidinopropane) dihydrochloride (AAPH) and treated with the extracts for 24 h. Figure 7 shows the normalized TEER values, which indicate a clear difference between the negative control and the AAPH-treated sample throughout the experiment. While the TEER value of the AAPH-treated cells steadily decreased and reached the minimum at approximately 15 h, the TEER value of the unstressed control remained stable for a longer time and did not drop below 50% of the starting value.

At approximately 2.5 h post-treatment, a clear increase in the TEER value was observed in the presence of *Protect* compared to AAPH. The values increased to levels greater than those of the unstressed control, remained constant for approximately 6 h, and then, dropped, but remained higher than those of the AAPH control. *ROI* combined with 5 mM AAPH slightly increased the TEER values compared to the AAPH control. Two representative time points (10 and 20 h post-treatment) were selected for significance analysis. At both time points, *Protect* significantly increased the TEER value compared to the stressed control. *ROI* ameliorated the decrease in TEER in response to AAPH treatment; however, the effect was not as pronounced as that of *Protect*.

### 2.7. Protect and ROI Extracts Improve Intestinal Integrity and Reduce Mortality Rates of D. melanogaster

**Figure 8:**
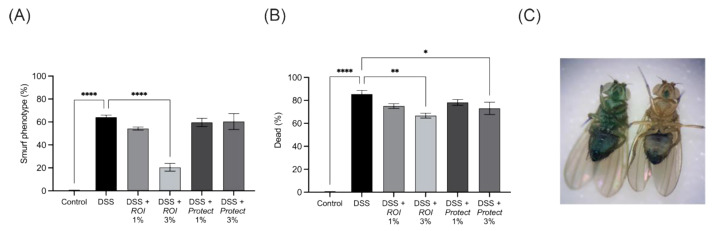
Mean fractions of Smurf flies (**A**) and the survival of experimental flies (**B**) after 7 days of DSS challenge. Comparison of dead flies with Smurf (left) and non-Smurf (right) phenotypes (**C**). (**A**,**B**) represent the pooled data of 3 DSS challenge experiments and the scoring of flies with the Smurf phenotype. The total n per treatment group was ~300 flies. Error bars are based on the SEM. *: *p* < 0.05, **: *p* < 0.01, ****: *p* < 0.0001.

To further study the effects of *ROI* and *Protect* extracts on the intestinal barrier, we used a *D. melanogaster* model challenged with dextran sodium sulphate (DSS). DSS is highly toxic to intestinal epithelial cells and compromises barrier function [41]. Smurf flies are used as models of leaky gut syndrome caused by DSS, as the Brilliant Blue FC dye is able to leak to surrounding tissues. A comparison of Smurf and non-Smurf flies is shown in Figure 8C. Treatment with 3% *ROI* significantly decreased the total number of Smurfs, as well as mortality, in DSS-challenged *D. melanogaster*, while 3% *Protect* did not affect the number of flies with the Smurf phenotype (Figure 8). However, *Protect* also decreased mortality compared to DSS alone. The lower extract concentration (1%) only reduced the mortality rate. In conclusion, based on in vitro and in vivo experiments, both wood lignan-containing extracts improved intestinal performance.

### 2.8. Impact of the Feed Supplement Protect on Piglets under Stress Conditions

**Figure 9:**
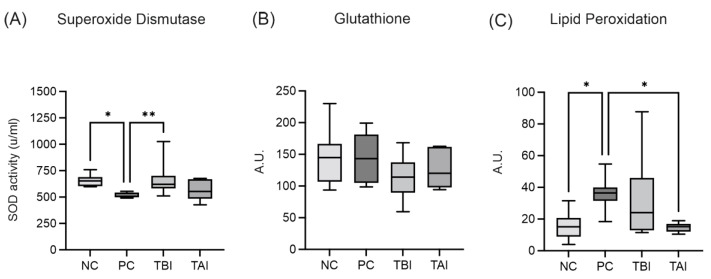
Quantitative analysis of superoxide dismutase (**A**), glutathione (**B**) and lipid peroxidation (**C**) in oxidative lesions recovered from the large intestine mucosa scrapings of piglets inoculated with *B. hyodysenteriae* and treated with *Protect* 7 days prior (TBI) or 3 days after (TAI) inoculation. The results are based on a minimum of 8 samples per group. NC: negative control; PC: positive control; TBI: treatment before inoculation; TAI: treatment after inoculation. *: *p* < 0.05, **: *p* < 0.01.

Based on our experimental data, as well as feedback from farmers, we studied the effects of *Protect* in a feeding trial. To assess oxidative injury between groups of piglets that were experimentally infected with *B. hyodysenteriae* and treated with *Protect*, oxidative lesions were recovered from large intestine mucosa scrapings and analyzed for the antioxidant markers glutathione (GSH) and superoxide dismutase (SOD), and lipid peroxidation (LPO; Figure 9 and Table S1). As shown in Figure 9A, the activity of SOD was significantly decreased in the positive control group compared to the negative control group that was not infected with *B. hyodysenteriae*. The addition of *Protect* to the feed starting 7 days before inoculation (TBI group) resulted in a significant increase in SOD activity. The addition of *Protect* 3 days post-inoculation (TAI group) also slightly increased SOD activity. No significant differences in GSH concentration were observed among the four experimental groups. LPO was significantly increased in the positive control group compared to the negative control group. LPO was significantly decreased in the TAI group and showed a decreasing trend in the TBI group.

**Figure 10:**
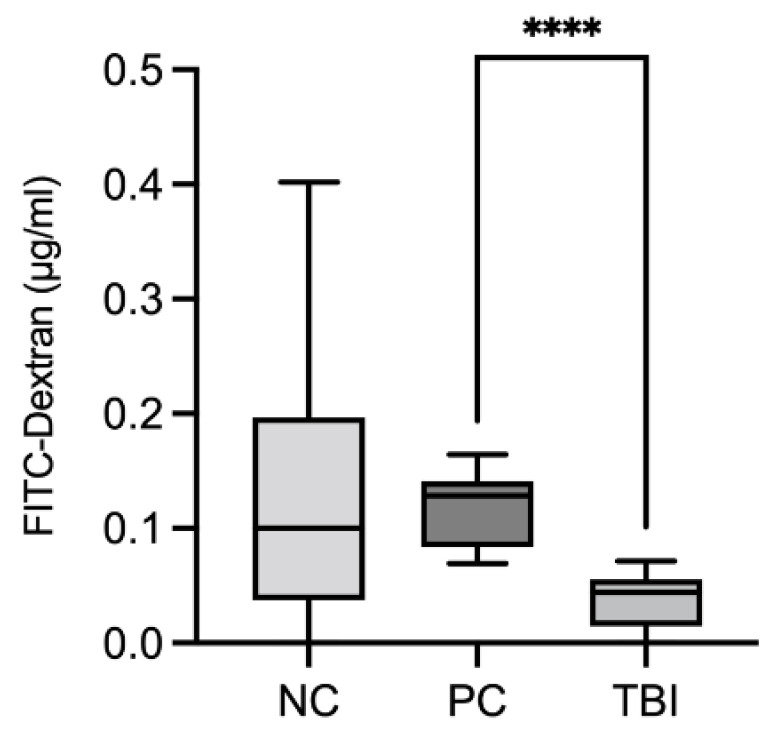
Comparison of FITC-dextran recovery among piglets that were experimentally inoculated with *B. hyodysenteriae* and treated with *Protect* starting 7 days prior to inoculation (TBI group). Six days post-inoculation, FITC-dextran was ingested orally, and after 6 h, blood samples were collected and analyzed. The results are based on 10 samples per group. NC: negative control; PC: positive control; TBI: treatment before inoculation. ****: *p* < 0.0001.

Intestinal permeability in piglets that were experimentally inoculated with *B. hyodysenteriae* and treated with *Protect* was assessed using the fluorescent marker fluorescein isothiocyanate (FITC)-dextran, which was recovered in the blood after oral administration. FITC-dextran is a molecule with a high molecular weight and is not permeable to the intact intestinal epithelium and cannot bind to plasma proteins. Six days post-inoculation, FITC-dextran was ingested orally, and after 6 h, blood samples were collected and analyzed. We hypothesized that infection with *B. hyodysenteriae* promoted inflammation and epithelial lesions and the accumulation of FITC-dextran levels in the blood due to the increase in epithelial permeability. Figure 10 and Table S2 show the comparison of FITC-dextran recovery between the three experimental groups. The addition of *Protect* to the feed 7 days before inoculation (TBI group) significantly reduced FITC-dextran recovery compared to the positive control group, indicating reduced intestinal permeability under these conditions.

## 3. Discussion

In this study, we investigated two wood lignan-based feed supplements (*Protect* and *ROI*) to determine their antioxidant and anti-inflammatory properties and their effects on intestinal barrier function. We provide evidence that lignans and soluble tannins derived from wood can support intestinal health by exerting antioxidant effects, inhibiting certain proinflammatory cytokines and strengthening barrier integrity.

The extracts prepared from *ROI* and *Protect* were first characterized by their wood lignan content. HPLC analyses revealed the presence of magnolol and honokiol in both extracts, with a slightly lower honokiol concentration in *Protect*. Additional unidentified compounds were found in both extracts, but especially in *Protect*. Importantly, these compounds possibly also contribute to the biological activity described here and need to be identified in future studies. However, the analysis of tannins is hampered by their complexity and the lack of reliable standards and quantitative analytical methods.

Next, the total phenolic content and antioxidant capacity of extracts prepared from *Protect* and *ROI* were analyzed, and both were elevated in *Protect* compared to *ROI*. This finding is consistent with the higher polyphenolic content of *Protect* due to additional supplementation with an extract from *Castanea sativa* that contains soluble tannins. Phenolic compounds are major contributors to the antioxidant activity of plant extracts, and the antioxidant capacity and total phenolic content, as measured using TPC and TEAC, are closely linked [42]. The antioxidant capacities of the *Protect* and *ROI* extracts were further investigated in the Caco-2 colorectal adenocarcinoma cell line. Pretreatment with *Protect* could significantly reduce AAPH-induced ROS generation, similar to the control quercetin. Because *ROI* did not significantly alter ROS levels, the antioxidant effect of *Protect* is likely related to its phenolic content. Phenolic compounds are generally known to scavenge ROS, as they can easily donate electrons to reactive radicals generated by AAPH [43]. In a similar study, Reggi and colleagues demonstrated that 3 h of pretreatment with a wood extract containing high levels of polyphenols, especially tannins, could counteract H_2_O_2_-induced oxidative stress in the intestinal IPEC-J2 cell line [36]. The antioxidant activity of *Protect*, determined in this study using TEAC and ROS assays, could be mediated by the high levels of soluble wood tannins.

Next, the effects of *ROI* and *Protect* extracts on the immune response were investigated in THP-1 macrophages. Gut-associated lymphoid tissue is the largest immune organ of the body, providing the first line of defense against pathogens and developing immune tolerance against commensal gut microbiota and nonharmful food components. In addition to playing a central role in preventing host infection, macrophages that migrate into the laminar propria play an important part in maintaining intestinal tissue homeostasis. In particular, intestinal tissue-resident macrophages can reduce inflammation and regulate enterocyte proliferation, as well as wound repair [44]. The THP-1-cell line is a valuable model to study the immune response capacity of monocytes and macrophages. When treated with phorbol 12-myristate 13-acetate (PMA), these cells differentiate into macrophage-like cells, which behave similarly to native monocyte-derived macrophages [45,46]. In our study, the mRNA expression levels of the proinflammatory cytokines IL-8, IL-6, and TNFα were significantly diminished by the addition of *ROI* extract in the cell culture medium in comparison to the control. *Protect* extract significantly reduced the mRNA expression of only TNFα. Cytokine secretion analysis revealed significantly reduced concentrations of IL-6 and TNFα in the cell culture supernatant when the cells were treated with *ROI* compared to the LPS control. In contrast, the IL-8 concentration was not reduced, although IL-8 mRNA expression was downregulated by *ROI*, and while IL-1β expression was not significantly altered, the supernatant level was considerably reduced by *ROI*. The mRNA expression of the cytokines was measured 24 h after treatment, but cytokine levels in the supernatant accumulated over the entire treatment period. Therefore, these results may not be directly comparable. However, the results suggest that *ROI* exhibits anti-inflammatory activity by inhibiting proinflammatory cytokine expression and secretion in LPS-stimulated macrophages. These results are partially in agreement with previous studies conducted on the anti-inflammatory effects of magnolol and honokiol. Lee et al. reported a significant reduction in IL-8 and TNFα concentrations in the supernatant of challenged THP-1 cells following 24 h treatment with 10 µM honokiol and magnolol [47]. Using *ROI* and *Protect* extract concentrations of 0.05 and 0.005%, the lignan content in our study was remarkably lower, which might explain why IL-8 secretion was not reduced in our experiment. Additionally, Lee and colleagues used undifferentiated THP-1 cells challenged with *Propionibacterium acnes*, whereas in our study, differentiated THP-1 cells were stimulated with LPS, making a direct comparison difficult. However, *ROI* similarly reduced TNFα concentration, which could be a result of the higher honokiol content compared to *Protect*. Honokiol (1-20 µM) has previously been shown to significantly reduce the LPS-induced expression and secretion of IL-6, IL-1β and TNFα in endometrial endothelial cells (bEEC), similar to the *ROI* extract in our study [48]. By using LPS-stimulated mouse uterine epithelial cells, Luo and colleagues demonstrated that the inhibition of IL-6 and TNFα production in response to magnolol treatment was due to the downregulation of Toll-like receptor 4 (TLR4) expression, which attenuated TLR4-mediated NF-κB and mitogen-activated protein kinase (MAPK) signaling, thus inhibiting the production of these proinflammatory cytokines [49]. The downregulation of IL-1β, TNFα and IL-6 by magnolol has also been reported in rats challenged with LPS [50]. The anti-inflammatory effects of wood-derived polyphenols have not been well described in the literature, although some of them have been reported to inhibit NF-κB signaling and IL-1β production in macrophages [51,52] and mice [53,54]. The reduction in proinflammatory cytokine production by *ROI* is likely related to its levels of magnolol and honokiol. Another possible reason for the reduced anti-inflammatory effect of *Protect* compared to *ROI* might be the low concentration used in the experiment. This concentration was used to avoid the potential cytotoxic effects of *Protect* on THP-1 cells, as the 0.01% extract induced a notable decrease in cell viability, which was not the case for *ROI*.

We also analyzed the effects of the *ROI* and *Protect* extracts on the intestinal barrier function of a Caco-2 cell layer and of the model organism *D. melanogaster* under stressed conditions. *In vitro*, noninvasive TEER measurements allowed for the continuous monitoring of monolayer integrity after the application of the stressor AAPH. The addition of *Protect* reversed the AAPH-induced weakening of barrier integrity, and *ROI* was less effective than *Protect*. A lower *ROI* concentration was used, as preliminary data revealed drastic TEER lowering in response to higher concentrations, indicating cytotoxic effects on the monolayer.

The effects of the *ROI* and *Protect* extracts on intestinal function were further evaluated in flies. Here, higher concentrations were used to assess intestinal permeability because they showed no toxicity in preliminary experiments. *D. melanogaster* is a strong model for investigating intestinal health and is often preferred over mammalian models. The intestinal structural organization and functions of *D. melanogaster* share many relevant similarities with mammalian gastrointestinal systems. The posterior midgut of *D. melanogaster* is highly metabolically active and immune-responsive and corresponds to the human small intestine. The literature indicates that immunometabolic pathways that maintain intestinal integrity, homeostasis and functions are particularly similar between *D. melanogaster* and humans [55].

In our experiment, 3% *ROI* extract strongly reduced the fraction of Smurf flies challenged by DSS, indicating the protective effects of *ROI* on intestinal barrier function. Leakage of Brilliant Blue FCF dye to the hemolymph and all tissues in Smurf flies is a result of barrier dysfunction initiated by the stressor DSS [56]. Since the loss of barrier integrity causes death in *D. melanogaster* [57], *ROI* could significantly reduce the mortality rate. *Protect* also reduced the mortality rate, albeit not as much as *ROI*.

The use of different concentrations in experiments using Caco-2 cells and *D. melanogaster* could account for the differing effects of *Protect* and *ROI* on these two model systems. To the best of our knowledge, no prior studies have examined the impact of wood-derived polyphenols and lignans on the intestinal barrier integrity of a cell layer or the model organism *D. melanogaster*; however, animal feeding trials have already been conducted in this context. Recent research on mouse models has provided indications of the ability of these wood-derived compounds to strengthen the intestinal barrier. By increasing the expression of epithelial tight junction proteins, restoring intestinal villus height and crypt depth, and inhibiting colon length reductions, magnolol could prevent gastrointestinal toxicity induced by oxaliplatin in mice [32]. Deng and colleagues reported comparable protective effects of magnolol and honokiol by using a diarrhea mouse model [29]. Similarly, pretreatment with wood-derived tannins could improve intestinal barrier functions in mice [58]. These effects have also been observed in farm animals. The supplementation of feed with magnolol and honokiol (300 mg/kg) for 12 weeks improved gut health in laying hens by significantly increasing jejunum and ileum villi length and increasing the mRNA expression levels of the tight junction proteins zonula occludens-1 and claudin-1 [59]. The same effect of magnolol has been reported in broiler chickens [60]. Wood-derived tannins exert similar improvement effects on intestinal barrier function in broiler chickens. Liu and colleagues showed that supplementation of the chicken diet can counteract the negative effects of heat stress, as indicated by significantly reduced serum D-lactate concentrations and diamine oxidase activity—which reflect the integrity of the small intestinal mucosa—as well as increased mRNA levels of zonula occludens-1 [40]. These reductions in diamine oxidase activity due to tannin supplementation have also been observed in weaned piglets, suggesting improvements in intestinal barrier integrity and function [61,62].

Given the promising observations in Caco-2 cells and in *D. melanogaster*, we conducted a feeding trial with piglets that were experimentally infected with *B. hyodysenteriae* to evaluate the effect of wood-based feed supplements on intestinal barrier integrity and antioxidant capacity. *Protect* was preferred over *ROI* in the feeding trial because of its enrichment in soluble tannins. *B. hyodysenteriae* is the primary etiologic agent of swine dysentery, a severe mucohemorrhagic colitis in pigs that is characterized by bloody and watery diarrhea, dehydration and reduced growth. Lesions are limited to the colon and may progress to marked inflammation with excessive mucus production, necrosis and hemorrhage [63,64]. Colitis caused by *B. hyodysenteriae* infection in growing pigs has been suggested to be associated with compromised colonic gut barrier functionality through the loss of epithelial cells or the disruption of tight junctions [65]. We assessed intestinal permeability in piglets infected with *B. hyodysenteriae* and supplemented with *Protect* (1.5 kg/t) starting 7 days before infection by measuring the FITC-dextran recovery rate in the blood after oral administration. High serum FITC-dextran recovery indicated increased intestinal permeability due to disrupted barrier integrity. In our study, there was no significant difference in FITC-dextran recovery between the NC and PC groups, possibly because of the high variability in the NC group. This finding was also observed in a recent study by Helm and colleagues, who showed that *B. hyodysenteriae* infection in growing pigs was not necessarily associated with reduced intestinal integrity and increased permeability [66]. We could, however, show that the addition of *Protect* to the feed 7 days before infection in the TBI group substantially decreased the FITC-dextran recovery rate compared to that in the positive control group, indicating that pretreatment with *Protect* improved intestinal barrier integrity.

Additionally, we analyzed oxidative injury in piglets that were experimentally infected with *B. hyodysenteriae* and supplemented with *Protect* 7 days before inoculation or 3 days after inoculation. Oxidative lesions recovered from large intestine mucosa scrapings were examined to determine the antioxidant markers SOD, GSH and LPO. Piglets infected with *B. hyodysenteriae* in the PC group showed significantly lower SOD activity than those in the NC group. Treatment with *Protect* before infection significantly increased SOD activity, highlighting its protective antioxidant effect, while only a slight, nonsignificant effect was observed after post-infection treatment. No significant differences in GSH concentration were observed in the four experimental groups. LPO was significantly elevated after infection, and post-treatment with *Protect* in the TAI group reduced LPO levels. Thus, *Protect* had some antioxidant effects on infected piglets, as indicated by increased SOD activity and decreased LPO levels. To our knowledge, no previous study has examined the impact of wood-based feed additives, especially lignans in combination with soluble tannins, on oxidative injury in piglets infected with *B. hyodysenteriae*. Recently, increased serum SOD and GSH levels were observed in healthy ducklings following supplementation with magnolol [67]. A similar study reported increased GSH and SOD levels in the breast muscle and jejunum of healthy broilers in response to magnolol treatment [68]. The same increases in GSH and SOD have been observed in mice [69]. While these studies reveal the in vivo antioxidant activity of wood-derived lignans or phenolic compounds, no information has been provided on the combination of these two substances. In this study, we demonstrated that combined dietary supplementation with lignans and soluble tannins from wood could improve barrier function and antioxidant status in the intestines of piglets infected with *B. hyodysenteriae*.

## 4. Materials and Methods

### 4.1. Extract Preparation

The wood lignan-based products *ROI* and *Protect* were obtained from Agromed Austria GmbH (Kremsmünster, Austria). Both products are feed supplements for poultry and pigs. According to the available product specification, *ROI* appears as a fine granulate of white to light-grey color and contains lignocellulose, calcium carbonate, silica and zinc chloride. *Protect* appears as a fine powder of dark brown color and contains lignocellulose, silica and zinc chloride. *Protect* is additionally complemented by a blend of extracts from the bark of *Castanea sativa*, obtained via a physical extraction process, and contains polyphenols, especially soluble tannins.

For extract preparation, 5 g of the products *ROI* and *Protect* were mixed with 30 mL of 1% acetic acid (*v/v*). After thorough mixing and sonification, extraction was carried out in a thermoshaker (37 °C, 4 h). Following centrifugation (RT, 10 min, 4600 rpm), the supernatant was transferred to a 50 mL volumetric flask. The residue was slurred with several portions of 1% acetic acid using the ultrasonic bath, and centrifuged again. The supernatants were collected in the volumetric flask and filled up to a defined volume of 50 mL. The extraction was carried out in duplicate in each case. These extracts were used for all in vitro analyses and for *D. melanogaster* experiments.

### 4.2. Determination of the Main Lignans Contained in ROI and Protect Extracts via HPLC

Extract analyses were performed using reversed-phase chromatography, as described previously [70], using a Thermo Scientific Dionex Ultimate 3000 comprised of a LPG-3400SD pump with built-in degasser, a WPS-3000 U(T)SL cooled autosampler, a temperature-controlled column compartment and an FLD-34000RS diode array detector (DAD) equipped with the Chromeleon software. Analyte separation was performed on an Accucore C18 column (150 mm × 3.0 mm inner diameter, 2.6 μm particle size; Thermo Scientific). The column temperature was set to 40 °C and the injection volume was 1 μL for standard calibration and 1 or 10 µL for sample measurement. The ultraviolet (UV) wavelength was detected at 260 nm. The analytes were separated via gradient elution with mobile phase A containing 0.1% formic acid (FA) in water and mobile phase B containing 0.1% FA in acetonitrile at a flow rate of 0.5 mL/min. The elution gradient starting conditions were 95% A and 5% B. After 5 min of equilibration time, the proportion of B was increased to 20% at 8 min and to 40% at 12 min, followed by 60% B at 15 min and 80% B at 17 min for 3 min. B was reduced to 5% at 20 min until 25 min. The lignan standards honokiol and magnolol were purchased from Extrasynthese (Genay, France). Both were dissolved in DMSO to a defined volume with 50% acetonitrile/water, and a standard series was prepared. For the samples, identification was performed using the retention time and UV spectrum. Evaluation was performed using Chromeleon software (Chromeleon 7.2.10 Thermo Scientific).

### 4.3. Determination of TPC and TEAC

The extract *Protect* was diluted in distilled water at a ratio of 1:1000. The extract *ROI* was used undiluted. Before usage, the extract samples were centrifuged at 13,600 rpm for 10 min at RT. A total of 100 µL extract sample was then mixed with 1 mL distilled water and 100 µL Folin–Ciocalteu (FC) reagent. After an incubation step of 6 min at room temperature, 500 µL saturated sodium carbonate solution was added to the sample. This was followed by an incubation period of 70–75 min in the dark at RT. A total of 200 µL of the sample mixtures, the blank and standards were then pipetted into a 96-well plate in triplicate. The plate was incubated for 75 min in the dark at room temperature. After incubation, absorbance was measured at 750 nm using a microplate reader (POLARstar Omega, BMG Labtech, Ortenberg, Germany). The TEAC assay measures the antioxidant capacity of a given substance compared to the standard, Trolox. Most commonly, antioxidant capacity is measured using the ABTS (2,2’-azino-bis(3-ethylbenzothiazoline-6-sulfonic acid))-decolorization assay. The ABTS solution (7 mM, in potassium persulfate) was diluted 1:80 with distilled water. The extract *Protect* was diluted in distilled water at a ratio of 1:1000. The extract *ROI* was used undiluted. The diluted samples were centrifuged at 13,600 rpm for 10 min. A total of 200 µL of the ABTS work solution and 4 µL extract sample were pipetted into a 96-well plate. The plate was incubated for 5 min at room temperature. Absorbance was then measured at 734 nm using the POLARstar Omega microplate reader. For better comparability, the results were normalized to a dry sample weight. All reagents used were obtained from Merck KGaA (Darmstadt, Germany, Sigma Aldrich).

### 4.4. Cell Culture

The Caco-2 human epithelial (DSMZ, Braunschweig, Germany) cell line was cultured under conditions of 37 °C, 5% CO_2_ and humidified air in Eagle Minimum Essential Medium (EMEM + Earle’s Balanced Salt Solution + 2 mM L-glutamine + non-essential amino acid + 1 mM sodium pyruvate + 1.5 g/L sodium bicarbonate), supplemented with 10% fetal bovine serum (FBS) and 1% penicillin-streptomycin (P/S) (PAN-Biotech, Aidenbach, Germany). The cells were subcultured two times a week at splitting ratios between 1:3 and 1:5, depending on the confluency. For this, the Caco-2 cells were washed with phosphate-buffered saline (PBS) without calcium and magnesium (PAN-Biotech), detached using trypsin-EDTA solution (Biochrom GmbH, Berlin, Germany) and neutralized in fresh EMEM. For barrier integrity analysis, differentiated Caco-2 cells were used. A differentiated Caco-2 cell monolayer exhibits characteristics similar to absorptive enterocytes such as brush border and microvilli structures, the expression of tight junctions and typical enzymes and transporter systems [71]. Differentiation was achieved via cultivation in Enterocyte Differentiation Medium (Biocoat Inc, Horsham, PA, USA) for 72 h. While differentiated Caco-2 cell monolayers are applied to study intestinal functionality in vitro, undifferentiated Caco-2 cells are usually used to examine the anticancer or antioxidant activity of active substances [72]. Consequently, determination of the antioxidant activity of *ROI* and *Protect* extracts was performed in undifferentiated Caco-2 cells.

The THP-1 (DSMZ) cell line was cultured under conditions of 37 C, 5% CO_2_ and humidified air in RPMI 1640 Medium (+ 2 mM L-glutamine + 1 mM sodium pyruvate + 10 mM HEPES + 4.5 g/L glucose + 1.5 g/L sodium bicarbonate) (PAN-Biotech), supplemented with 10% FBS, 1% P/S and 0.05 µM 2-mercaptoethanol (Sigma Aldrich). The cells were subcultured two times a week via centrifugation with subsequent resuspension at 1.5–2 × 10^5^ cells/mL. To induce differentiation into a mature macrophage-like state, the THP-1 monocytes were treated with 50 ng/mL PMA (Sigma Aldrich) for 48 h. Consequently, the suspended monocytes were differentiated into adherent macrophages. Successful differentiation was defined by an adherence rate of ~90%. All cell culture experiments with the THP-1 cell line were performed in differentiated THP-1 cells.

### 4.5. Determination of Cell Viability in THP-1 and Caco-2 Cells

Cytotoxicity was measured using the CellTiter-Glo luminescent cell viability assay (Promega Corporation, WI, USA). CellTiter-Glo solution was prepared according to the manufacturer’s instructions. The Caco-2 cells were seeded into black 96-well plates (Greiner Bio-One, clear bottom) in quadruplicate at 7.5 × 10^5^ cells per mL and grown for 24 h (200 µL/well). For differentiation, the Caco-2 cells were seeded into black 96-well plates (Greiner Bio-One, clear bottom) in groups of six at 8.25 × 10^5^ cells per mL and grown overnight (200 µL/well). Following this, the medium was replaced with 200 µL Enterocyte Differentiation Medium, supplemented with 0.1% serum extender (Corning Inc., NY, USA) and 1% P/S. The cells were incubated for 72 h. The THP-1 cells were seeded into quadruplicate into black 96-well plates (Greiner Bio-One, clear bottom) at a concentration of 5 x 10^5^ cells/mL (200 µL/well) and 50 ng/mL PMA was added to induce the differentiation process; then, they were incubated for 48 h. The outer wells were filled with 200 µL medium to avoid evaporation. After growth/differentiation, the medium was removed from the wells. The THP-1 cells were washed once with 200 µL PBS containing calcium and magnesium (PAN Biotech). For the experiments, the extracts were diluted in the medium (+ 1% P/S + 1% FBS), and then, added to the cells at 100 µL/well. The cells were incubated for 4 h (Caco-2) or 24 h (THP-1). After the incubation time, 100 µL/well of CellTiter-Glo solution was added and mixed by pipetting. The plate was then incubated at room temperature for 10 min. Luminescence was analyzed using the POLARstar Omega microplate reader. Cell viability was expressed relative to the untreated control.

### 4.6. Determination of Antioxidant Capacity in Caco-2 Cells

The in vitro antioxidant activities of the two extracts were measured by determining intracellular ROS using the cell-permeable reagent H_2_DCF-DA (Sigma Aldrich) as described in [73] with minor modification. The Caco-2 cells were seeded into black 96-well plates in triplicate at 1.5 × 10^5^ cells per well (200 µL/well) and grown overnight. On day two, the cells were co-treated with 100 µL extract dilutions or 20 µM quercetin (Sigma Aldrich) as a control, plus 50 µM H_2_DCF-DA or medium (as a non-fluorescent background control) at 37°C for 20 min. Then, the cells were washed with 200 µL Hank’s Balanced Salt Solution (HBSS) (Pan Biotech) and treated with 100 µL of the free-radical-generating compound AAPH (500 µM; Sigma Aldrich), or with HBSS as a control. The amount of DCF formed was determined by measuring with the POLARstar Omega microplate reader in fluorescence mode at 485 nm excitation and 530 nm emission wavelengths immediately after the addition of AAPH and every 30 min for 1.5 h. The DCF fluorescence intensity was background-corrected and normalized to the unstressed control. The slopes between the timepoints were then calculated assuming a linear signal increase, and normalized to the stressed control containing AAPH.

### 4.7. Determination of Inflammatory Gene Expression and Cytokine Concentration in THP-1 Cells

The THP-1 cells were seeded in duplicate into 6-well plates (Greiner Bio-One) at a concentration of 5 × 10^5^ cells/mL (5.5 mL/well) with 50 ng/mL PMA to induce the differentiation process. The cells were incubated for 48 h. After the incubation time, the medium was carefully withdrawn from the wells and the cells were washed once with 3 mL of PBS containing calcium and magnesium. The extracts were diluted in the medium (+ 1% P/S, + 1% FBS) to 0.05% (for *ROI*) or 0.005% (for *Protect*), and 250 ng/mL LPS (Sigma Aldrich) was added as stressor. The cells were treated with 2.5 mL of the extract dilutions and incubated for 24 h. The following day, the supernatants were collected, centrifuged (200 g, 4 min, room temperature) and frozen in 3 × 500 µL aliquots at −80 °C for cytokine secretion analysis. The cells were lysed, and the RNA was isolated using the QIAGEN RNeasy Plus kit (Qiagen, Hilden, Germany) according to the manufacturer’s instructions. The RNA concentrations were measured using the POLARstar Omega microplate reader. The RNA samples were stored at −80 °C until RT-qPCR analysis.

The mRNA expression levels of the genes IL-1β, IL-6, IL-8 and TNFα were measured quantitatively via RT-qPCR (C1000 Thermal Cycler and CFX96 Real-Time System, Bio-Rad Laboratories, Vienna, Austria). A total amount of 50 ng of RNA was reverse-transcribed into cDNA using the iScript cDNA Synthesis Kit, and RT-qPCR with the iQ SYBR Green Supermix was carried out according to the manufacturer’s instructions (both from Bio-Rad Laboratories, Vienna, Austria). DNA denaturation and polymerase activation were performed for 3 min at 95 °C and followed by 40 PCR cycles. One amplification cycle was divided into three parts: denaturation at 95 °C for 10 s, annealing at 60 °C for 30 s and extension at 72 °C for 10 s, with a plate read after each cycle. The gene expression of the target genes in each experiment was normalized to the expression of two reference genes, namely glyceraldehyde-3-phosphate dehydrogenase (GAPDH) and ribosomal protein L5 (RPL5). In all RT-qPCR analyses, the detected C_T_ values were used to calculate the mRNA expression levels via the 2^-∆cT^ method [74]. The oligonucleotide sequences of the primers (Microsynth AG, Balgach, Switzerland) used are shown in Table 1.

Secretion of the inflammatory cytokines IL-1β, IL-6, IL-8 and TNFα in LPS-stimulated THP-1 cells was quantified using a Luminex xMAP^®^ custom 4-plex assay (Bio-Techne Sales Corp., Abingdon, UK) according to the manufacturer’s instructions. The cell culture supernatants were diluted 1:100 and 1:2000 for IL-1β, IL-6, TNFα and IL-8 detection, respectively. In brief, 50 µL of the sample or standard and 50 µL precoated MagPlex microbeads were incubated for 2 h, followed by washing and coating with 50 µL biotinylated antibodies. After 1 h of further incubation, the microbeads were washed, and Streptavidin-PE solution was added for 30 min. Finally, the beads were washed once again and resuspended in 100 µL washing buffer. All incubation periods occurred in a sealed, light-protected black-bottom plate using a thermoshaker at RT, 800 rpm. The prepared samples were measured using Luminex^®^ 200™ and analyzed using xPonent^®^ acquisition software, version 4.3 (both Luminex Corp., Austin, TX, USA).

### 4.8. Determination of Intestinal Barrier Integrity in Caco-2 Cells

The intestinal barrier integrity of the differentiated Caco-2 cell layers was determined using the CellZscope2 (nanoAnalytics GmbH, Münster, Germany), which measures the TEER of cell layers automatically. For this, 24-well plates (Cellstar, Greiner bio-one, Kremsmünster, Austria) were filled with 1040 µL/well of pre-warmed EMEM, supplemented with 10% FBS and 1% P/S. Inserts (ThinCerts^TM^, 0.4 µm, translucent, Greiner bio-one) were placed into the wells using sterilized tweezers. Subsequently, the Caco-2 cells were seeded into the inserts at a concentration of 5.5 × 10^5^ cells/mL (300 µL/insert). The cells were incubated for 24 h. Following this, the wells of the CellZscope2 were filled with 950 µL Enterocyte Differentiation Medium (Biocoat Inc.), and supplemented with 0.1% serum extender (Corning Inc.) and 1% P/S. The medium inside the inserts was then discarded, and the inserts were transferred from the 24-well plate to the CellZscope2. The cells were incubated for 72 h until a complete differentiation level and a stable TEER value were achieved. For the extract treatment, the basal and apical differentiation media were removed completely. The extracts were diluted in EMEM (- FBS, +1% P/S) with and without 5 mM AAPH and applied apically (300 µL/insert) and basolaterally (950 µL/well) in triplicate. The cells were incubated and the TEER values were documented for 24 h. The TEER values were normalized to the starting TEER value of each sample.

### 4.9. Determination of Intestinal Barrier Function in D. melanogaster

#### 4.9.1. *w^1118^* D. Melanogaster Rearing Conditions and Synchronization

The wild-type strain *w^1118^* of *Drosophila* *melanogaster* was kindly provided by Dr. Gerald Rimbach (University of Kiel, Kiel, Germany)—originally strain no. 5905 (Bloomington Drosophila Stock Center, Indiana University, Bloomington, IN, USA)—and reared in our laboratory for over 50 generations. Parental and experimental flies *w^1118^* were reared in a climate chamber HPP750 eco (Memmert GmbH + Co. KG, Germany) at 25 °C and 60% relative humidity with a programmed 12:12 h light-dark cycle. If not stated otherwise, all reagents in the *D. melanogaster* section were obtained from Carl Roth (Karlsruhe, Germany). For the egg collection, parental flies were transferred to embryo collection cages (Genesee Scientific, San Diego, CA, USA) with Nutri-Fly^®^ Grape Agar Petri dishes (Genesee Scientific) prepared according to the manufacturer instructions. For the first 24 h, the parental flies were supplemented with fresh yeast paste prepared with active dry yeast “Red star” (Genesee Scientific) and deionized water. Afterwards the Petri dish was discarded and replaced with a fresh one. Eggs were collected the next day and dispensed on modified Caltech medium. The larval medium was composed as described in [75] from 1% agar, 5.5% glucose, 3% sucrose, 2.5% inactive dry yeast, 6% yellow cornmeal (both Genesee Scientific), 1% of methyl 4-hydroxybenzoate solution (10% *w*/*v* solution in absolute ethanol) and 0.48% of 99% propionic acid. After 9 days, freshly enclosed synchronized adults were transferred to fresh stock bottles with sugar–yeast medium (1.5% agar, 5% sucrose, 10% yeast extract, 1% of methyl 4-hydroxybenzoate solution (10% solution in absolute ethanol, *w/v*) and 0.48% propionic acid) and allowed to mate for 2 days.

#### 4.9.2. Intestinal Barrier Challenge

A series of three experiments were conducted to investigate the protective effects of *ROI* and *Protect* during the DSS challenge of *D. melanogaster* as described by Amcheslavsky and colleagues with modifications [41]. Adult 5-day-old *w^1118^* females in groups of 25±2 per vial were transferred to vials containing 1.5% agar with a piece of 1.5 × 3 cm gel and 1.5 mm thick blotting paper (Whatman, Cytiva, UK) soaked with one of the experimental media consisting of 5% sucrose, 1% Brilliant Blue FCF (C.I. 4209), 5% DSS with an average MW of 40,000 g/mol, and 1% or 3% *ROI* or *Protect* aqueous solutions, respectively. The control group was fed with 5% sucrose and 1% Brilliant Blue FCF. Female flies were transferred to a fresh agar vial with a piece of filter paper with treatment solution at least four times per experiment. The sorted adult flies were maintained in standard climate conditions at 25 °C and 60% relative humidity for 7 days. The number of dead flies with and without the Smurf phenotype was recorded daily as described in [56,57]. Each experimental condition was represented by ~100 flies (4 vials × 25 females per condition per experiment). Only females with a well-expressed Smurf phenotype (blue coloration observed outside abdominal area, e.g., in thorax and limbs) were considered Smurfs.

### 4.10. Piglet Feeding Study Experimental Design

For the experiment, 40 21-day-old piglets were used, from a nucleus farm, with no clinical history of infections by *Brachyspira* spp., *Lawsonia intracellularis* or *Salmonella* sp. The animals were randomized and separated into four groups: a negative control group not inoculated with *B. hyodysenteriae* and not supplemented with the feed supplement *Protect* (NC) (*n* = 10), a positive control group inoculated with *B. hyodysenteriae* and not supplemented with *Protect* (PC) (*n* = 10), a group supplemented with *Protect* 7 days before inoculation with *B. hyodysenteriae* (TBI) (*n* = 10), and a group supplemented with *Protect* after inoculation with *B. hyodysenteriae* (TAI) (*n* = 10). All the piglets were acclimated to the facilities for 7 days, receiving feed and water ad libitum.

The pathogenic strain of *B. hyodysenteriae*, used to prepare the inoculum, was obtained in 2013 from a pig that had a severe clinical case of swine dysentery, from a farm located in the state of Minas Gerais. Cultivation was carried out as described by Leser et al. [76]. Briefly, the pathogenic strain of *B. hyodysenteriae* was sown on Trypticase Soy Agar (TSA) supplemented with 5% equine blood under an anaerobic atmosphere (80% N_2_, 10% CO_2_ and 10% H_2_) at 37 °C for three days. Then, TSA Agar plates were washed with sterile PBS. The PBS acquired with the washing of the plates was incubated in Brain Heart Infusion (BHI) growth broth enriched with 10% FBS and 0.2% sodium bicarbonate, adapted from Kunkle et al. [77], in a ratio of 1:100 mL (washed: broth), for 21 h at 39 °C in a shaker oven; subsequently, the animals were inoculated.

Seven days before inoculation, the animals in the TBI group began to receive feed supplemented with the solid feed additive *Protect* at a rate of 1.5 kg per ton of feed, until the end of the experiment. On day 0 of the experiment, 14 days after arrival, the animals in the TBI, TAI and CP groups were challenged, intragastrically using flexible gavage, for three consecutive days with 50 mL of inoculum containing an average of 5.31 × 10^8^ organisms of *B. hyodysenteriae*/mL per day. The animals in the negative control group received an equal volume of sterile BHI broth. The animals in the TAI group received feed supplemented with *Protect* from the third day after the first inoculation until the end of the experiment. Fourteen days after the first inoculation, all animals were euthanized and necropsied according to the criteria established by CEUA Animal Use Ethics Committee.

The present study was approved by the UFMG Animal Experimentation Ethics Committee at an online meeting on 06 June 2020 with protocol number 80/2020.

#### Oxidative Injury and Intestinal Permeability Assessment

Oxidative lesions were measured for the concentration of GSH, SOD and LPO; these were recovered from large intestine mucosa scrapings of all piglets, obtained from the apex of the spiral colon, on the day of euthanasia. The mucosal scraping samples were collected and immediately frozen in liquid nitrogen, soon after euthanasia and macroscopic evaluation, until the moment of analysis. The determination of LPO was carried out through the Fe^2+^ oxidation test in the presence of orange xylenol, known as the FOX test—Ferrous Oxidation/Xylenol [78]. The determination of SOD activity was performed using single-scan oscillopolarography [79]. The concentration of GSH was spectrophotometrically determined as described in [80].

In the analysis to assess changes in intestinal permeability, the recovery of serum dextran in the piglets was measured. Six days after the first inoculation, all piglets received, orally, a solution of FITC-dextran (1 mL) and, after 6 h, the blood was collected and centrifuged at 3780 g to obtain the serum. The serum samples were stored in liquid nitrogen until the moment of analysis. The levels of FITC-dextran in blood samples collected 6 days post-infection were determined by measuring fluorescence, with an excitation wavelength of 485 nm and an emission wavelength of 528 nm using a fluorescence spectrometer [81].

### 4.11. Statistical Analysis

Statistical analysis was performed using GraphPad Prism version 9.2.0 (GraphPad Software, San Diego, CA, USA). Outliers were identified via the ROUT method and excluded from the calculations. The statistical difference among means was determined using an ordinary one-way ANOVA and Šídák’s multiple comparison test or, if the standard deviations (SD) were significantly different, a Brown–Forsythe and Welch ANOVA test and Dunnett’s multiple comparison test. Figures were prepared using CorelDRAW 2019 (Corel Corporation, Ottawa, ON, Canada).

## 5. Conclusions

Preserving intestinal health is crucial for maintaining the overall health and performance of farm animals. In this study, we provide evidence that wood lignan-based feed supplements and extracts support intestinal health by improving barrier integrity and exerting antioxidant and anti-inflammatory activity. By using suitable in vitro and in vivo models, we demonstrated that lignans (magnolol and honokiol) and soluble tannins derived from wood inhibited the production of ROS, decreased the expression and secretion of certain proinflammatory cytokines and improved intestinal barrier function. Based on these observations, we evaluated the efficacy and the antioxidant and barrier-strengthening properties of the feed additive *Protect* in a feeding trial using piglets that were experimentally infected with *B. hyodysenteriae*. We showed, for the first time, that combined dietary supplementation with wood-derived lignans and soluble tannins could counteract the negative effects of *B. hyodysenteriae* infection on piglets by improving the antioxidant capacity and barrier integrity of the intestine. We conclude that secondary plant metabolites from wood exhibit promising potential for use in feed as natural intestinal health promoters and merit further research.

## Figures and Tables

**Table 1:** List of primers.

**Genes**	**Forward Primer Sequence (5’–3’)**	**Reverse Primer Sequence (5’–3’)**	**Accession No.**
IL-1β	CATGGGATAACGAGGCTTATG	ACAAAGGACATGGAGAACAC	NM_000576.3
IL-6	GACAGCCACTCACCTCTT	GGCAAGTCTCCTCATTGAATC	NM_000600.5
IL-8	CTGTGTGAAGGTGCAGTT	ACTTCTCCACAACCCTCT	NM_000584.4
TNFα	AGCACTGAAAGCATGATCC	GCCAGAGGGCTGATTAGA	NM_000594.4
GAPDH	TGGTATCGTGGAAGGACTCA	CAGTGAGCTTCCCGTTCAG	NM_002046.7
RPL5	TGGGCCAGAATGTTGCAGAT	AGGGACATTTTGGGACGGTT	NM_000969.5

## Data Availability

Data are available from the authors upon request.

## Supplementary Materials

The following supporting information can be downloaded at: https://www.mdpi.com/article/10.3390/molecules27196327/s1, Table S1: Comparison of SOD activity and GSH and LPO concentrations in oxidative lesions recovered from large intestine mucosa scrapings among groups of piglets experimentally inoculated with B. hyodysenteriae and treated with Protect starting 7 days pre (TBI)- or 3 days post (TAI)-inoculation. NC: negative control; PC: positive control; TBI: treatment before inoculation; TAI: treatment after inoculation. ns: *p* > 0.05, *: *p* < 0.05, **: *p* < 0.01. Table S2: Comparison of FITC-dextran recovery among groups of piglets experimentally inoculated with *B. hyodysenteriae* and treated with *Protect* starting 7 days pre (TBI)-inoculation. NC: negative control; PC: positive control; TBI: treatment before inoculation. ns: *p* > 0.05, ****: *p* < 0.0001.
