# Supplementary material for: Extracts Prepared from Feed Supplements Containing Wood Lignans Improve Intestinal Health by Strengthening Barrier Integrity and Reducing Inflammation
Source: Molecules. 2022 Sep 26;27(19):6327. doi: 10.3390/molecules27196327 (PMC9572150; doi:10.3390/molecules27196327)
Supplement: Supplementary file 1 [file molecules-27-06327-s001.zip › molecules-1908822-supplementary.pdf]

Supplementary Table S1 and S2

Table S1: Comparison of SOD activity and GSH and LPO concentrations in oxidative lesion recovered from large intestine mucosa scrapings among groups of piglets experimentally inoculated with *B. hyodysenteriae* and treated with *Protect* starting 7 days pre (TBI) or 3 days post (TAI) inoculation. NC: negative control; PC: positive control; TBI: treatment before inoculation; TAI: treatment after inoculation. ns: p > 0.05, *: p < 0.05, **: p < 0.01.

Marker	Group 1	Group 2	n 1	n 2	p value	Significance
SOD	NC	PC	10	8	0.0115	*
SOD	PC	TBI	8	10	0.0090	**
SOD	PC	TAI	8	10	0.7736	ns
SOD	TBI	TAI	10	10	0.0832	ns
GSH	NC	PC	10	9	>0.9999	ns
GSH	PC	TBI	9	10	0.2556	ns
GSH	PC	TAI	9	10	0.8149	ns
GSH	TBI	TAI	10	10	0.8327	ns
LPO	NC	PC	10	8	0.0217	*
LPO	PC	TBI	8	10	0.9739	ns
LPO	PC	TAI	8	8	0.0215	*
LPO	TBI	TAI	10	8	0.0531	ns

Table S2: Comparison of FITC-dextran recovery among groups of piglets experimentally inoculated with *B. hyodysenteriae* and treated with *Protect* starting 7 days pre (TBI) inoculation. NC: negative control; PC: positive control; TBI: treatment before inoculation. ns: p > 0.05, ****: p < 0.0001.

Group 1	Group 2	n 1	n 2	p value	Significance
NC	PC	10	10	0.9979	ns
PC	TBI	10	10	<0.0001	****
